# Genetic and Genomic Tools to Asssist Sugar Beet Improvement: The Value of the Crop Wild Relatives

**DOI:** 10.3389/fpls.2018.00074

**Published:** 2018-02-06

**Authors:** Filipa Monteiro, Lothar Frese, Sílvia Castro, Maria C. Duarte, Octávio S. Paulo, João Loureiro, Maria M. Romeiras

**Affiliations:** ^1^Centre for Ecology, Evolution and Environmental Changes, Faculdade de Ciências Universidade de Lisboa, Lisboa, Portugal; ^2^Linking Landscape, Environment, Agriculture and Food, Instituto Superior de Agronomia, Universidade de Lisboa, Lisboa, Portugal; ^3^Institute for Breeding Research on Agricultural Crops, Julius Kühn-Institut, Federal Research Centre for Cultivated Plants (JKI), Quedlinburg, Germany; ^4^Department of Life Sciences, Centre for Functional Ecology, Universidade de Coimbra, Coimbra, Portugal

**Keywords:** *Beta*, *Patellifolia*, crop wild relatives, crop breeding pool, next-generation sequencing

## Abstract

Sugar beet (*Beta vulgaris* L. ssp. *vulgaris*) is one of the most important European crops for both food and sugar production. Crop improvement has been developed to enhance productivity, sugar content or other breeder's desirable traits. The introgression of traits from Crop Wild Relatives (CWR) has been done essentially for lessening biotic stresses constraints, namely using *Beta* and *Patellifolia* species which exhibit disease resistance characteristics. Several studies have addressed crop-to-wild gene flow, yet, for breeding programs genetic variability associated with agronomically important traits remains unexplored regarding abiotic factors. To accomplish such association from phenotype-to-genotype, screening for wild relatives occurring in habitats where selective pressures are in play (i.e., populations in salt marshes for salinity tolerance; populations subjected to pathogen attacks and likely evolved resistance to pathogens) are the most appropriate streamline to identify causal genetic information. By selecting sugar beet CWR species based on genomic tools, rather than random variations, is a promising but still seldom explored route toward the development of improved crops. In this perspective, a viable streamline for sugar beet improvement is proposed through the use of different genomic tools by recurring to sugar beet CWRs and focusing on agronomic traits associated with abiotic stress tolerance. Overall, identification of genomic and epigenomic landscapes associated to adaptive ecotypes, along with the cytogenetic and habitat characterization of sugar beet CWR, will enable to identify potential hotspots for agrobiodiversity of sugar beet crop improvement toward abiotic stress tolerance.

## CWR for sugar beet improvement: current status and prospects

Sugar beet (*Beta vulgaris* L. ssp. *vulgaris*, cultivar group Sugar Beet) is one of the most important crops, being within the 2013 top 10 world commodities, with Europe contributing with 68% production (Food Agricultural Organization of the United Nations, 2016). The sugar beet accounts for 20% of the global sugar production. The genus also includes the cultivar groups Fodder Beet, Garden Beet, and Leaf Beet (Lange et al., 1999). The origin and domestication of sugar beet have been comprehensively reviewed in Biancardi et al. (2012). As one of the youngest crops, sugar beet breeding pool was considered narrow, limiting breeding progress (Bosemark, 1979). Since then, *B*. *vulgaris* L. ssp. *maritima* L. (Arcang.) was extensively used as a source of resistance gene (Biancardi et al., 2012) to specifically complement the breeding pool. At the global market sugar beet competes with sugar cane. In order to stay competitive an incremental breeding progress is no longer sufficient. Rather, a performance leap is required as targeted by the French AKER project (AKER, 2017). To this end, today's sugar beet breeding pools need to be purposefully broadened by incorporating alleles from wild species which may have gone lost during the domestication history of cultivated beets or have never existed in the breeding pool due to crossing barriers between the wild ancestor of cultivated beets and related wild species (Frese et al., 2001).

A taxonomic system allowing reliable inferences on the phylogeny, the geographic spread of species during evolution and the today's genetic relationships between taxa facilitates the identification of genetic resources suitable for base broadening programs. The taxonomic inconsistencies within the subfamily Betoideae have been reported (e.g., Ford-Lloyd, 2005; Hohmann et al., 2006) repeatedly (see Table 1). While the taxonomy of *Beta* section *Beta* is settled, uncertainties still exists with respect to section *Corollinae* and the genus *Patellifolia* (former *Beta* section *Procumbentes*) (Frese, 2010; Frese et al., 2017).

**Table 1:** Classifications of the subfamily Betoideae (Amaranthaceae).

	**Classifications of the subfamily Betoideae**				Distribution of *Beta* and *Patellifolia* species^*^
**Tribe**	**Ulrich (****1934****)**	**Ford-Lloyd (****2005****)**	**Hohmann et al. (****2006****)**	**Romeiras et al. (****2016****)**	
**Beteae**	***Beta***	***Beta***	***Beta***	***Beta***	
	sect. *Corollinae*	sect. *Beta*	sect. *Beta*	sect. *Beta*	Western Mediterranean region and Macaronesian archipelagos *B. vulgaris* ssp. *maritima* (L.) Arcang—Mediterranean coasts (Iberian Peninsula and North Africa), Azores, and Madeira *B. macrocarpa* Guss.—Iberian Peninsula, North Africa and Canaries *B. patula* Aiton —Madeira (endemic) *B. vulgaris* ssp. *vulgaris*—cultivated
	sect. *Nanae* sect. *Procumbentes*	sect. *Corollinae* sect. *Nanae*	sect. *Corollinae* (incl. sect. *Nanae*)	sect. *Corollinae* (incl. sect. *Nanae*)	Eastern Mediterranean region and Southwestern Asia *B. corolliflora* Zosimovic ex Buttler *B. intermedia* Bunge ex Boiss. *B. lomatogona* Fisch. and C.A.Mey. *B. mac*rorhiza Steven *B. trigyna* Waldst & Kit. *B. nana* Boiss and Heldr.
	sect. *Vulgares*	sect. *Procumbentes*		*Patellifolia*	Western Mediterranean region and in Macaronesian archipelagos *P. procumbens* (C.Sm.) A.J.Scott, Ford-Lloyd and J. T.Williams—Madeira, Canaries and Cabo Verde (endemic) *P. webbiana* (Moq.) A.J.Scott, Ford-Lloyd and J. T.Williams—Canaries (endemic) *P. patellaris* (Moq.) A.J.Scott, Ford-Lloyd and J. T. Williams—Iberian Peninsula, Italy, North Africa, Madeira, Canary and Cabo Verde
**Hablitzieae**	*Acroglochin*	*Acroglochin*	*Patellifolia*		
	*Aphanisma*	*Aphanisma*	*Aphanisma*	*Aphanisma*	
	*Hablitzia*	*Hablitzia*	*Hablitzia*	*Hablitzia*	
	*Oreobliton*	*Oreobliton*	*Oreobliton*	*Oreobliton*	

* For details see Romeiras et al. (2016)

The widespread use of genetically uniform crop varieties has caused agricultural crops to lose some of the genetic diversity present in their wild progenitors. CWR offer important sources of useful agronomic traits, including: intermediate C3-C4 photosynthetic activity; tolerance for cold, salt and drought conditions, and nutraceutical characteristics, i.e., plant-based compounds with health-protective roles. (Zhang et al., 2017). Selection almost inevitably causes unintentional loss of genetic diversity in the breeding pools. However, as long as breeding pools can be replenished by introgression or incorporation of genetic diversity contained in wild species, the genetic diversity in breeding programs can be kept in balance as was discussed by Ordon et al. (2005). Since loss of genetic diversity in breeding pools as well as genetic erosion in CWR within their natural habitats are both slow and long-term processes, the connection between breeding progress in crop species and the need for effective conservation programs for CWR tends to be overlooked. Clearly, without CWR conservation programs operative within the next 10 years future breeding progress will be at risk. Genetic and genomic tools already support planning of CWR conservation and effective breeding programs as exemplified by Andrello et al. (2017). Their investigations provided insight into the geographic patterns of genetic diversity, which is not only relevant for CWR conservation planning but also contributed to the understanding of statistical relations between genetic markers and environmental variables.

The West Mediterranean Region encloses a number of undisturbed habitats (e.g., cliff coasts, and salt marshes) that holds some of the most important CWR of sugar beet, namely the sea beet (*B. vulgaris* ssp. *maritima*) and other endemic species within *Beta* (*B. macrocarpa* and *B. patula*), as well as *Patellifolia* (*P. patellaris, P. procumbens, P. webbiana*). Considering the available yet unexplored wild germplasm from *Beta* and *Patellifolia* species occurring in this region, their potential for supplementing sugar beet breeding pool is easily recognized, mainly due to their occurrence in habitats of extreme conditions. Generally, crop yield reduction is a consequence of increasingly abiotic stresses (Mickelbart et al., 2015), which is a major limiting factor in plant growth. Indeed, drought is expected to cause salinization of 50% of all arable lands by 2050 (Ashraf and Wu, 1994). Although sugar beet breeding programs have already allowed the introgression of genes related to disease resistance from wild *Beta* and *Patellifolia* species (e.g., Munerati, 1932; Gidner et al., 2005), through marker-assisted crossing (Francis and Luterbacher, 2003), work on abiotic tolerance still remains underdeveloped. Therefore, the genetic characterization of traits responsible for the adaptation of wild populations to saline and/or hot and dry habitats should be a viable step toward raising the level of abiotic stress tolerance in sugar beet breeding pools.

In this perspective, we pointed out that some genetic and genomic tools are presently available for screening for trait variation. In this way wild species of *Beta* and *Patellifolia* could be explored to uncover novel variation in functional traits associated to adaptive capacity under abiotic stresses.

## Establishing the relatedness between crops and CWR

To ascertain the degree of relatedness between CWRs and crops, several schemes have been proposed. Harlan and de Wet (1971) suggested an informal classification system and assigned species to the primary (GP1), secondary (GP2) and tertiary (GP3) genepool using the strength of crossing barriers between the crop species and wild species as criteria. The tertiary genepool describes the extreme outer limit of the potential genepool of a crop. If information on the reproductive isolation is lacking the “Taxon Group concept” of Maxted et al. (2006) can be applied and the taxonomic hierarchy be used to assess the relatedness between the crop and wild species potentially suited as gene donors. More recently, Vincent et al. (2013) defined the “provisional gene pool concept” (PGP) as *to be used when there is no formally published gene pool concept and when taxonomic treatments lacked subgeneric information, but there is published crossability evidence between the crop and related taxa*. Thus, determination of genetic diversity allied to taxonomy is of major interest when using CWR, as both genetic distance and species classification can be assigned.

In the face of environmental changes likely resulting in a dramatic loss of CWR in Europe (Aguirre-Gutiérrez et al., 2017), the understanding of the relationships among taxa of agronomically important crops is not of marginal interest (Knapp et al., 2013). It is of crucial importance as breeders should be enabled to capture genetic diversity present in CWR before they get lost and to maintain the transferred valuable traits in breeding pools.

Classification within Betoideae has been frequently altered, which challenges the assignment of CWR taxa to a gene pool. Recently, a phylogeny reconstruction of this subfamily (Romeiras et al., 2016), pointed out that *Patellifolia*, formerly included in *Beta* section *Procumbentes*, should be a separate genus, supporting that genetic divergence is responsible for the crossing difficulties faced in breeding programs. An early diversification between *Beta* (GP1, GP2) and *Patellifolia* (GP3) is postulated, and within GP1 and GP2 an ecological divergence between West and East Mediterranean *Beta* species was identified (Romeiras et al., 2016). Also, Frese et al. (2017) assessed genetic diversity in *P. patellaris* revealing that occurrences from Portugal are genetically different from the Spanish ones (Andrello et al., 2017), thus highlighting that Portuguese populations may harbor a different genetic variation that could be associated to the restricted coastal areas where they occur. Several genetic diversity studies in sea beet (e.g., Leys et al., 2014; Andrello et al., 2016) showed a distribution of genetic diversity according to ecogeographical ranges and, recently, discrimination in Portuguese populations from dissimilar habitats were accomplished (Ribeiro et al., 2016). Overall, studies with sea beet populations occurring in West Mediterranean Region point out to a clinal gradient, thus promoting adaptive radiation into ecoclines of populations from GP1 species (Monteiro et al., 2013). The main outcomes of phylogenetic and genetic diversity studies suggest that agronomically important traits associated to abiotic stress reside in wild species of the GP1 and GP3 and could be used to broaden the genetic basis of sugar beet.

## Agrigenomics: CWR as important sources

**Figure 1:**
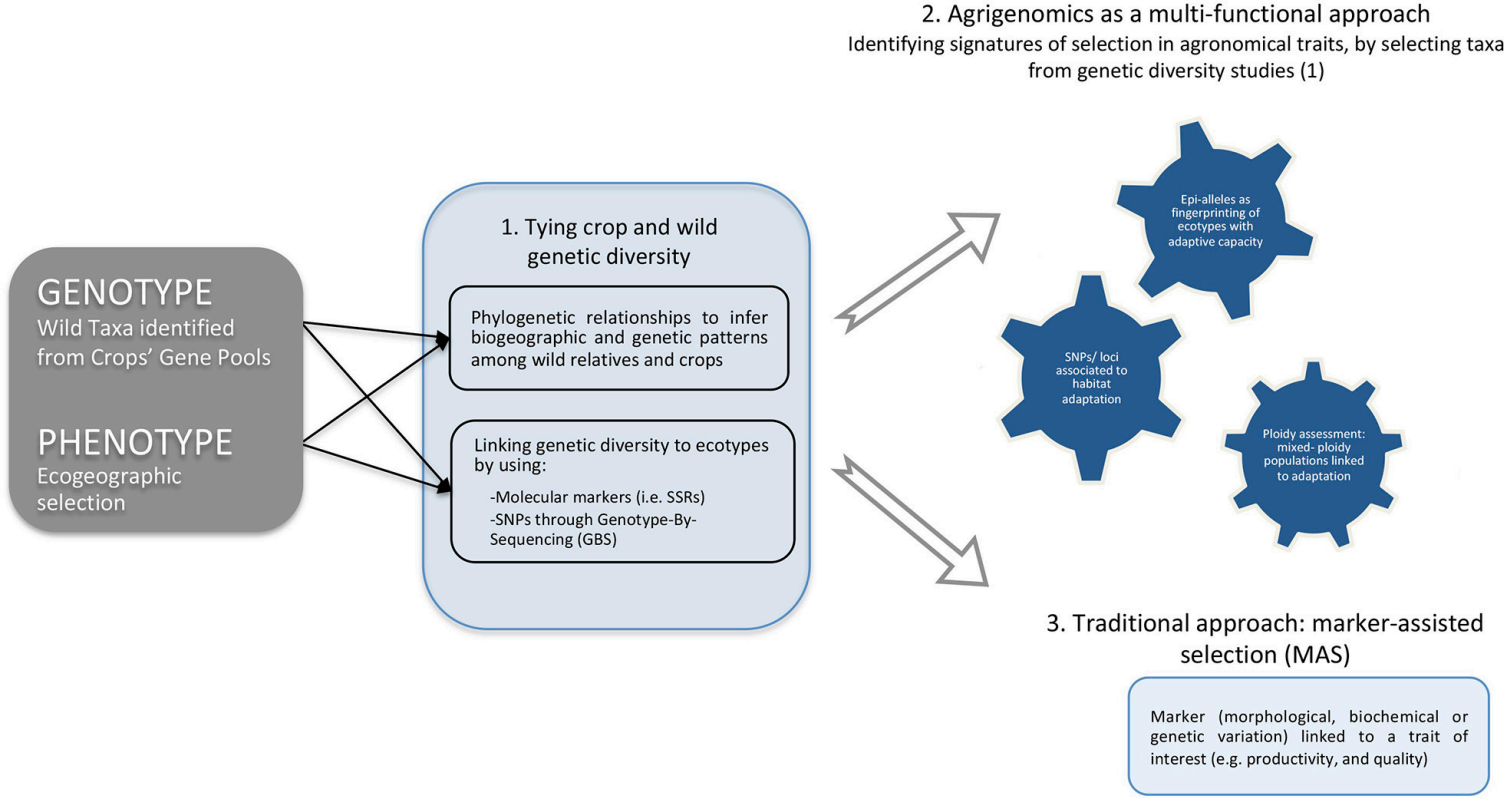
Genetic and genomic tools streamline toward a proposed genomic-assisted breeding strategy using wild relatives. Genotype and phenotype interactions, through identifying wild taxa from crop's gene pools and by selecting taxa occurring in different ecogeographic, should be considered the first step prior to any genetic and/or genomic prospection. From genetic studies (1), the determination of the genetic diversity between crop and wild relatives are the key to assess the relatedness of wild taxa with the crop itself, either by phylogenetics or by assessing genetic diversity with ecological ranges using high-resolution power molecular markers, e.g., microsatellites (SSRs) or SNPs through Genotype-By-Sequencing (GBS) approaches. The agrigenomics approach (2) is hereby proposed as multi-functional method to identify signatures of selection in agronomical traits, by selecting taxa from genetic diversity studies (1), rather than using neutral markers with are not subjected to selection. Thus, by selecting SNPs/Epi-alleles associated to adaptive capacity of extreme habitats in wild taxa along ploidy assessment, it will be possible to detect genetic variation potential on adaptive ecotypes on wild relatives of crops. Particularly, agronomic traits can be disclosed from genes/function/epigenomics/ploidy assessments toward the utilization in future crop improvement as a genomics-assisted breeding approach. Conversely, the traditional approach (3) only allow to incorporate a marker (morphological, biochemical or genetic variation) linked to a trait of interest (e.g., productivity, and quality) using marker-assisted introgression, thus not taking into consideration the complete genomic panorama need to understand the adaptive capacity of a plant that could be transferable effectively to a crop.

Before CWR can be used in any plant-breeding program, only if genetic variation in traits of interest for breeders is evaluated they are included as genetic resources. The field of Agrigenomics is in the focus of a technological revolution caused by the emergence of high-throughput DNA sequencing technologies. Recent studies highlight the importance of prospecting CWRs and crops with the current advances in genome sequencing (Bevan et al., 2017). For example, a 43% reduction in genetic diversity in modern maize lines was reported when compared to their progenitor populations (Wright et al., 2005); sequencing of 31 wild and cultivated soybean genomes identified higher allelic diversity in wild accessions (Lam et al., 2010). Altogether, these studies indicate a loss of genetic diversity caused by a genetic bottleneck during the domestication process. The completion of the sugar beet genome sequencing provided genomic resources to support molecular breeding (Dohm et al., 2014). However, identification of agronomic traits linked to adaptive phenotypic capacity, through assessment of *in situ* CWR populations that occur under abiotic stresses, should be an important follow up for finding “new genetic variation” which may benefit sugar beet breeding (Figure 1).

### Recovering the diversity lost by domestication

Through the relationship between genetic factors and phenotypes, a genomics-assisted breeding will be possible to assist onto the sustainable production at global food needs. Thus, identifying adaptive variation from neutral mutations is an important feature toward the understanding of the molecular basis of heritable phenotypic traits. Rather than genome sequencing alone, the reduction of the complexity of a genome by the Genotyping by sequencing (GBS), allows a high-throughput sequencing approach of multiplexed samples that associates genome-wide molecular marker discovery and genotyping (see He et al., 2014). Large-scale discovery of single nucleotide polymorphisms (SNPs) through restriction enzymes -site associated DNA (RAD) sequencing, have been successfully applied in crop genetics for ascertain markers linked to disease resistance (e.g., Talukder et al., 2014).

A recent study used 200 naturally occurring sea beets, to identify the sugar beet resistance gene *Rz2* with a modified version of mapping-by-sequencing (Capistrano-Gossmann et al., 2017). This study features the prominent potential of CWR for rapid discovery of causal genes relevant for crop improvement. Several studies in wild and domesticated crops using GBS tools underpinned the potential of wild populations from diverse agro-climatic regions for genetic enhancement of adaptive traits in crop gene pools (e.g., Bajaj et al., 2015) and distinct traits of cultivated and wild accessions associated to domestication process (e.g., Yang et al., 2016; Marrano et al., 2017). By using a whole genome SNPs approach on wild relatives of crops it would be possible to identify naturally selected trait-regulating genomic targets/functional allelic variants associated to adaptive capacity for genetic enhancement of cultivated gene pools. Specifically, by identifying signatures of selection in *Beta* and *Patellifolia* species that occur in ecotypes under drought/salt conditions, genomic information behind adaptive capacity could be assessed.

### Epialleles as fingerprinting of CWR

As sessile organisms, plants develop several mechanisms to cope with abiotic and biotic stresses. Besides heritable phenotypic variation within a species, phenotypic plasticity is considered one of the major means by which plants can cope with environmental factor variability (Boyko and Kovalchuk, 2011; Zheng et al., 2017). Epigenetic modifications are thought to play a particularly important role in fluctuating environments (Kooke et al., 2015), in contrast to DNA sequence variation. Epigenetics refers to meiotically or mitotically heritable variations of phenotypic traits caused by genetic modifications, especially DNA methylation (Ekblom and Galindo, 2011). Epigenetic variations with stability over multiple generations have been reported in processes of local adaptation (Dubin et al., 2015). In plant systems, epigenetic inheritance is well documented (Taudt et al., 2016), and epigenomic variation at a locus can be treated as a quantitative trait. Long-term exposure to abiotic and biotic conditions shape distinct heritable epigenetic landscapes (e.g., Feil and Fraga, 2012), thus major differences in epigenetic landscapes are expected when comparing distinct ecotypes (Flatscher et al., 2012). As epigenetic variation can be environmentally induced, this source of natural variation in ecologically relevant traits may be subjected to selection (Latzel et al., 2013). Former studies identified epigenetic variation as being responsible for phenotypic plasticity in mangrove individuals [*Laguncularia racemosa* (L.) C.F.Gaertn] occurring in distinct habitats (Lira-Medeiros et al., 2010). Despite the importance on characterizing epigenetic landscapes across ecological ranges (Rodríguez López and Wilkinson, 2015), little information is available outside of model organisms (Fortes and Gallusci, 2017), and particularly in natural populations. The potential of epigenetics to play a role in crop improvement is growing, namely by the identification/selection of epialleles (Springer, 2013). New sequencing tools as bisulfite-converted RADseq (BsRADseq), an approach to quantify the level of DNA methylation differentiation across multiple individuals (Trucchi et al., 2016), allow an epigenomic screening in natural populations. Large-scale epigenetic surveys will allow comparison of epigenetic variation in natural *Beta* and *Patellifolia* species, occurring in extreme habitats, and their association to phenotypic variation could be addressed. In this context, investigating the extent of epigenetic divergence from natural *Beta* and *Patellifolia* populations that thrive in different ecological conditions, would allow to determine the heritable epigenetic landscapes shaped by abiotic conditions. As such, epialleles identified would be an innovative tool useful as an epi-fingerprinting for selecting resilient sugar beet genotypes, which can better cope with environmentally challenging conditions. The development of new breeding strategies that could incorporate epigenomic information is a major challenge. Epigenome editing tools as CRISPR/Cas9 have been considered a promising tool for targeted epigenetic-marker breeding strategies by selecting agronomical desirable quantitative traits (Thakore et al., 2016).

### Cytogenomics

In breeding programs, the importance of interspecific hybridization and polyploidy has long been widely acknowledged (Mason, 2016). Crops can cross-pollinate with their related wild species and exchange chromosome segments by homoeologous recombination. Such hybrids are most often sterile, but chromosome doubling (either spontaneous or instantaneously, originating allopolyploids) or the fixation of viable recombinant chromosome sets (homoploidy) can help to overcome hybridization barriers, obtain sterile cultivars and restore fertility in hybrids (Rieseberg and Carney, 1998). Polyploidy can also contribute to enhanced pest resistance (Heijbroek et al., 1983) and stress tolerance (Colmer et al., 2006) and/or enhanced crop vigor (Nassar et al., 2008). In sugar beet, polyploid breeding was also used to increase crop yield (Jusubov, 1967; Xuan et al., 2009). These allopolyploid or homoploid forms can constitute important bridges and gene reservoirs for subsequent gene flow back to their diploid progenitors (Benavente et al., 2008).

Nowadays, different cytogenomic techniques, from classical cytogenetic methods, cytomolecular approaches (including different fluorescent *in situ* hybridization—FISH), such as the use of different types of DNA probes, from repeated DNA sequences and BAC clones to microdissected chromosomes, pachytene spreads, extended DNA fibers, among others—(Benavente et al., 2008) to flow cytometry can be used to study genomes. These techniques enable to distinguish genomes, identify specific regions in the chromosomes, and/or detect chromosome doubling.

In the *Beta-Patellifolia* species several cytogenetic studies have been developed. The section *Beta* has been described as cytogenetically uniform, mostly harboring diploid species. However, the detection of tetraploid individuals of *B. macrocarpa* in wild populations from the Canary Islands (Buttler, 1977), clearly revealed the need for wide geographical studies that could attest the cytogenetic diversity within the wild *Beta*. Indeed, Castro et al. (2013) revealed a cytogenetically diverse scenario. The authors analyzed several wild *Beta* populations across mainland Portugal and islands, and although most of the studied populations were diploid, they also discovered novel cytogenetic diversity. In particular, both diploid and tetraploid individuals were found in one population of *B. vulgaris* ssp. *maritima*, and *B. macrocarpa* revealed even more diversity with two populations harboring two or three cytotypes, including diploids and tetraploids, and/or hexaploids, the later described for the first time (Castro et al., 2013). These populations bearing cytogenetic diversity are of major importance for conservation and genetic resources management programs. The tetraploid *Beta macrocarpa* has been suggested to have an allopolyploid origin, resulting from hybridization between *B. vulgaris* ssp. *maritima* and diploid *B. macrocarpa* (Villain et al., 2009). Interestingly, previous works in Californian populations have documented the occurrence of hybridization between *B. vulgaris* and *B. macrocarpa*, showing introgression of *B. vulgaris* alleles into the later species (Bartsch and Ellstrand, 1999). The genus *Patellifolia* currently recognize three species, but still presents several taxonomic problems that need to be solved. Species boundaries have been questioned by several authors. For example, the diploid species were observed hybridizing spontaneously in natural populations and could form fertile offspring (Szota, 1964/1971; cited in Jassem, 1992), raising questions on if they should be treated as variants of the same species. Later, Wagner et al. (1989) also questioned if the diploids *P. procumbens* and *P. webbiana* were distinct species. The genus *Patellifolia* also revealed to have cytogenetic diversity. Giménez and Cueto (2009) studied *P. patellaris* from Andalucía and described it as a species having both diploid and tetraploid individuals. Recent analyses (unpublished data) confirmed these results, with *P. patellaris* being mainly tetraploid, while *P. procumbens* and *P. webbiana* being diploid. However, the cytogenetic diversity in certain regions/taxa was higher than expected: the ploidy of *P. patellaris* was variable with diploids being found in southeastern Spain and mainland Portugal. Also, in Tenerife, *P. patellaris* and *P. procumbens* co-occurred and seemed to cross and form a hybrid swarm, as supported by the occurrence of diploid, triploid and tetraploid plants and by the high morphological diversity. These results indicate that cryptic diversity and interspecific hybridization generates novel genetic variation within the genus, which benefits species survival as it may broaden the adaptive potential and also generate genetic variants of interest to plant breeding. The possible presence of cryptic diversity may also explain why the delineation of the three species is a challenge to genetic resources collectors and genebank curators.

Considering the importance of CWR for supplementing crops gene pool, species conservation actions in geographical regions encompassing mixed-ploidy populations, as recently reported in the *Beta-Patellifolia* species complex (Castro et al., 2013; unpublished data) could benefit plant breeding.

## Final remarks

In conclusion, we presented how the application of genomic tools could help uncovering new traits in CWR and how such diversity can be disclosed using high-throughput methodologies to identify new genomic information for breeding application. Such innovative tools will provide crucial genetic/epigenetic/cytogenetic elements to breeding programs. From identification to breeding application is a challenging step and will likely benefit from the emergence of genomics-breeding approach that is still in its infancy.

## Author contributions

FM and MR conceived the manuscript. All the authors improved upon successive versions.

### Conflict of interest statement

The authors declare that the research was conducted in the absence of any commercial or financial relationships that could be construed as a potential conflict of interest. The reviewer CB and handling Editor declared their shared affiliation.
